# Supplementary material for: Long-range correlation in protein dynamics: Confirmation by structural data and normal mode analysis
Source: PLoS Comput Biol. 2020 Feb 13;16(2):e1007670. doi: 10.1371/journal.pcbi.1007670 (PMC7043781; doi:10.1371/journal.pcbi.1007670)
Supplement: S1 Appendix — Detailed descriptions of the structural datasets involved in this research. Additional information concerning the scaling relations, generation of polymer structures, and other variations of elastic network models are also included in the Supplementary Information. (PDF) [file pcbi.1007670.s001.pdf]

# Supplementary Information of
Long-range Correlation in Protein Dynamics: Confirmation by Structural Data and Normal Mode Analysis

Qian-Yuan Tang, Kunihiko Kaneko

## 1 Datasets: Protein Structures determined by X-ray Crystallography and Nuclear Magnetic Resonance (NMR)

### 1.1 Protein Structures Determined by X-ray Crystallography

In the main text, our analysis is based on the protein structures determined by X-ray crystallography. As introduced in the main text, our dataset (Dataset A) contains 13081 proteins determined by X-ray crystallography. The PDB codes and the chain length of the proteins in Dataset A are listed in the file *"S1_PDB_Xray.txt"*. The chain length distribution of the proteins in Dataset A is shown in Fig.A(a).

### 1.2 Protein Structures Determined by Solution NMR

Here, in the Supplementary Information, another dataset (Dataset B) is introduced to compare with the proteins determined by X-ray crystallography. Dataset B contains 5078 proteins (no DNA, RNA or hybrid structures) determined by solution nuclear magnetic resonance (NMR). For every protein structure in Dataset B, there are no less than 4 different models describing the structure of such a protein. In Dataset B, every two proteins share less than 30% sequence similarity. The PDB codes and the chain length of the proteins in Dataset B are listed in the file *"S2_PDB_NMR.txt"*.

The chain length distribution of the proteins in Dataset B is shown in Fig.A(b). It is worth noting that the solution NMR is employed in determining the structures of small proteins, most of the proteins in Dataset B have chain length $N < 200$.

## 2 Fractal Dimensions of the Proteins

As discussed in the main text, there are accumulating evidence suggesting that the folded proteins have a fractal nature [1, 2, 3]. With the 3D coordinate of all the atoms in a protein molecule (PDB file), to determine the fractal dimension of the protein, one should first draw a sphere with radius $r$, then calculate the number of residues $n$ which are located within the sphere. As the radius $r$ increases, the number of residues $n$ located inside the sphere also increases. To describe such a dependence, the function $n(r)$ is introduced to quantify the packing of residues. For a 3D dense packing system, it is obvious that $n(r) \sim r^3$. Generalizing such a relation to $d$-dimensional systems, one can conclude that $n(r) \sim r^d$. For every protein molecule, by doing linear fitting for the log-log plot of $n(r)$ vs. $r$, the fractal dimension $d$ can be obtained.

The histogram of the fractal dimension is shown in Fig.A(c). For proteins determined by X-ray crystallography, the average fractal dimension $d \approx 2.7$. Such a result is consistent with the fractal dimension obtained by scaling analysis. For proteins determined by NMR, the average fractal dimension $d \approx 2.4$. Such a result indicates that the proteins in the solution are packed in a lower dimension than the crystallized proteins.

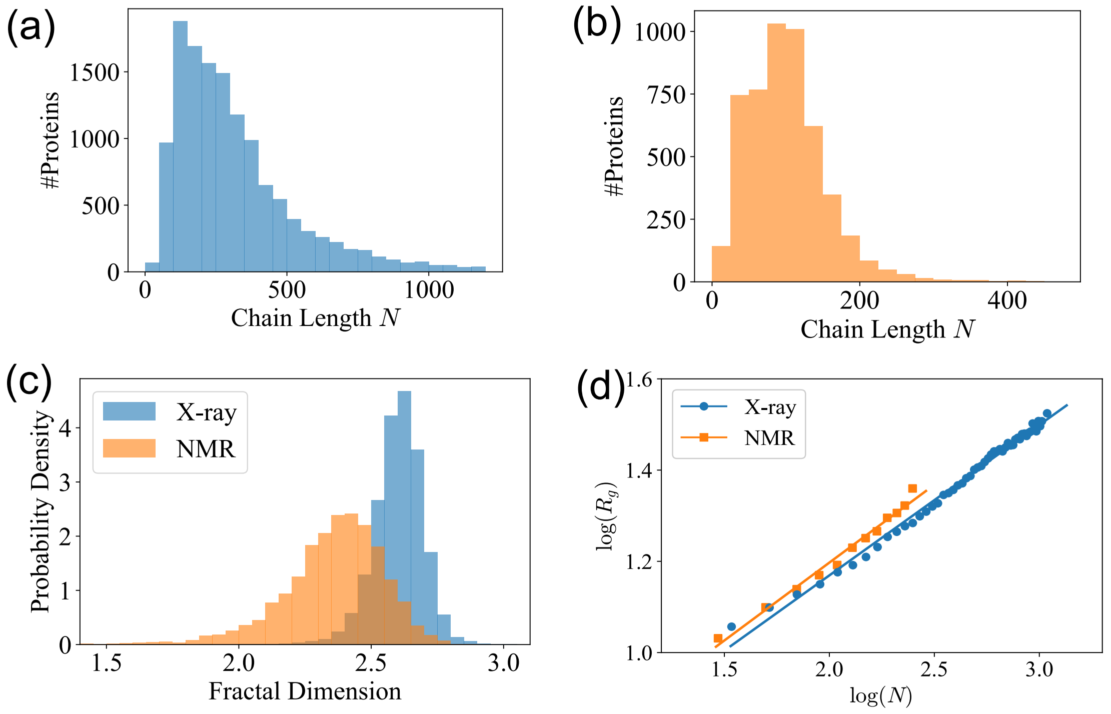


**Figure A. The statistics and scaling relations of native proteins in the datasets.** (a) The chain length distribution of the proteins in Dataset A (X-ray crystallography). In the histogram, the width of each bin is 50. (b) The chain length distribution of the proteins in Dataset B (NMR). In the histogram, the width of each bin is 25. (c) The fractal dimension distribution of the proteins determined by X-ray crystallography and NMR. (d) The log-log plot of the radius of gyration $R_g$ vs. chain length $N$.

## 3 Radius of Gyration

For the folded proteins, even the local packing of residues shows a fractal nature (with fractal dimension $d < 3$), the radius of gyration $R_g$ vs. chain length $N$ still behaves like dense packing systems. The proteins determined by both X-ray crystallography and NMR have the relation between $R_g$ and $N$: $R_g = r_0 N^{\alpha}$, where $r_0$ is a characteristic length quantifying the packing of amino acid residues. As shown in Fig.A(d), for proteins determined by NMR, $r_0 = 3.269$Å, and $\alpha = 0.341$; for proteins determined by X-ray crystallography, $r_0 = 3.233$Å, and $\alpha = 0.330$.

## 4 The Scaling Relations in Protein Systems

Previously, based on the NMR-determined ensemble, it was observed that there are plenty of scaling relations in the native fluctuations of the native proteins [4]. In the main text, based on the elastic network models of the proteins, the scaling relations of the proteins determined by X-ray crystallography are observed. Here, all the scaling relations and the values of the scaling coefficients are listed in the table below. The values in the brackets are obtained by the scaling analysis based on the NMR-determined structures [4].

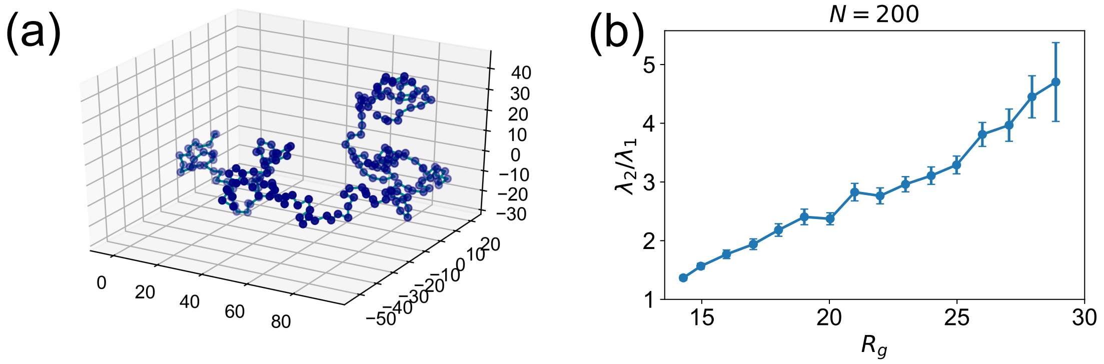


**Figure B. The structure and spectrum of the generated polymer chains.** (a) The structure of a generated freely-jointed chain with $N = 200$ beads $R_g = 36.02$Å. (b) The gap between the slowest and the second slowest eigenvalue for random polymer chains with different $R_g$. Here, all the polymer chains have $N = 200$ beads. In the figure, the error bars denotes for the standard error.

| **Scaling Relations** | **Scaling Coefficients** |
|---|---|
| $R_g \sim \xi \sim N^{\alpha}$ | $\alpha \approx 1.$ |
| $R_g \sim (s - s_C)^{-\nu}$ | $(\nu \approx 1/3.)$ |
| $\chi \sim (s - s_C)^{-\gamma}$ | $(\gamma \approx 1.)$ |
| $s - s_C \sim N^{-\alpha/\nu}$ | $(\alpha/\nu \approx 1.0.)$ |
| $\chi \sim N^{\alpha\gamma/\nu}$ | $\alpha\gamma/\nu \approx 1.$ |
| $\lambda_1 \sim N^{-\zeta}$ | $\zeta \approx 1.$ |
| $Q \sim 1 - K \cdot N^{-\eta}$ | $\eta \approx$ 0.231. |

## 5 Generating the Structures of Ideal Polymers

In the main text, we have compared the spectrum of proteins with the polymers. Here in this Supplementary Information, the detailed information of the generation of ideal (freely-jointed) polymer is provided. The generation of polymer structures follows the method described by Flechsig [5].

To generate the structure of the freely-jointed polymer, firstly, the position of the first bead is fixed at point $(0, 0, 0)$. Then, the next bead is placed at a random position near the previous bead. For every bead $i$, there are three restrictions for the preceding bead $i + 1$:

- The distance $r_{i,i+1}$ from bead $i$ to the preceding bead $i + 1$ have to lie within the interval between $d_{\min}$ and $d_{\max}$. In our calculation, we take parameters $d_{\min} = 4$Å, and $d_{\max} = 5$Å.
- The preceding bead $i + 1$ have to be separated from all previous beads $(1, 2, 3 \cdots, i)$ by at least the distance $d_{\min}$.
- The distance from preceding bead $i + 1$ to the geometric center of all previous beads should not exceed $r_{\max}$.

According to the restrictions listed above, the polymer chains can be generated. In our computation, a vary large $r_{\max}$ is selected to avoid strong spatial constraints. For a polymer chain that made up of $N = 200$ beads, we take $r_{\max} = 200$Å. In such a situation, the polymer chains generated can be recognized as a random walk without other kinds of interactions among beads. Such a model is called the freely-jointed chain (or ideal chain) model [6]. The structure of a freely-jointed polymer with $N = 200$ beads is illustrated in Fig.B(a).

A similar method can be introduced to generate polymer chains with spatial constraints. With different $r_{\max}$, one can generate polymer chains at different radius of gyration $R_g$. We have generated polymer chains with $N = 200$ beads, but with radius of gyrations $R_g$ ranging from 14

Å to 30. With the generated polymer structure, the vibration spectrum of a polymer chain is obtained based on the elastic network model. But it is worth noting that there are no "native states" for a random polymer chain in the real world. As shown in Fig.B(b), as the $R_g$ increases, the gap between the slowest and the second slowest eigenvalue (quantified by $\lambda_2/\lambda_1$) also increases.

# 6 Distance-dependent Force Constants

In the main text, we had conducted our analysis based on the simplest version of the Gaussian network model. Our correlation analysis and scaling analysis methods can also be extended to other versions of elastic network models. In real applications, by introducing distance-dependent force constants, refined models were suggested to have better predictions on the B-factors or the dynamics of proteins. Among the refined elastic network models, we select the Harmonic $C_\alpha$ potential model (HCA) [7, 8] and the parameter-free Gaussian network model (pfGNM) [9] to compare with the simplest form of GNM.

**HCA Model**. In HCA model, the force constant $\kappa_{ij}$ between residue $i$ and $j$ is defined as:

$$\kappa_{ij} = \begin{cases} a_1 r_{ij}^0 - b, & \text{if } r_{ij}^0 < c \quad (1) \\ a_2 (r_{ij}^0)^{-6}, & \text{if } r_{ij}^0 \geq c \quad (2) \end{cases}$$

where $r_{ij}^0$ denotes the equlibrium distance between residue $i$ and $j$, and parameter $c$ is set to 4Å so that the interactions between two sequential-neighboring $C_\alpha$ atoms are considered to be different from other interactions. Other parameters ($a_1$, $a_2$ and $b$) are fitted from experimental data.

**PfGNM**. In pfGNM, the force constants by the inverse-square of the equilibrium distance between the interacting nodes, that is:

$$\kappa_{ij} = (r_{ij}^0)^{-2}. \quad (3)$$

In the computation, one can also introduce other decaying exponent $p$, so that $\kappa_{ij} = (r_{ij}^0)^{-p}$. When $p = 6$, then the model would be very close to HCA model.

As shown in Fig.C(a) and C(c), with HCA model [7, 8], similar scaling relations ($\lambda_1$ vs. $N$, and $\chi$ vs. $N$) as discussed in the main text can also be observed. However, with pfGNM ($p = 2$), correct scaling relations cannot be reproduced. As shown in Fig.C(b) and C(d), by decreasing the interaction range (increase parameter $p$), the scaling coefficients gradually approach the correct value. Moreover, as shown in Fig.C(e) and C(f), pfGNM gives a much shorter correlation length. That is to say, although pfGNM can have good predictions of crystallographic B-factors, it fails to capture the long-range correlations and other important information in the solvated dynamics of protein molecules.

# 7 The Robustness of Topological Descriptors

In the main text, scaling relations are observed in the relation between the topological descriptors (average path length $\langle l \rangle$ and modularity $Q$) and the size of the proteins. It is worth noting that the corresponding critical exponents are very robust to the changes in the cutoff distances. In the main text, we select $r_C = 8$Å. As shown in Fig.D(a) and Fig.D(b), with different cut-off distances $r_c$, the coefficient in the scaling relation between the average path length $\langle l \rangle$ (or modularity $Q$) and chain length $N$ keeps as a constant, showing that the topological analysis conducted in the main text is robust to parameter changes.

# 8 Supporting Information: Long-range correlations in the flexibility of proteins

In X-ray crystallography, the B-factors (or temperature factors) is introduced to measure the deviations from an ideal crystal at zero temperature. Crystal disorder, finite-size effects, and thermal fluctuations can all contribute to the B-factors. In previous studies, the B-factors of the

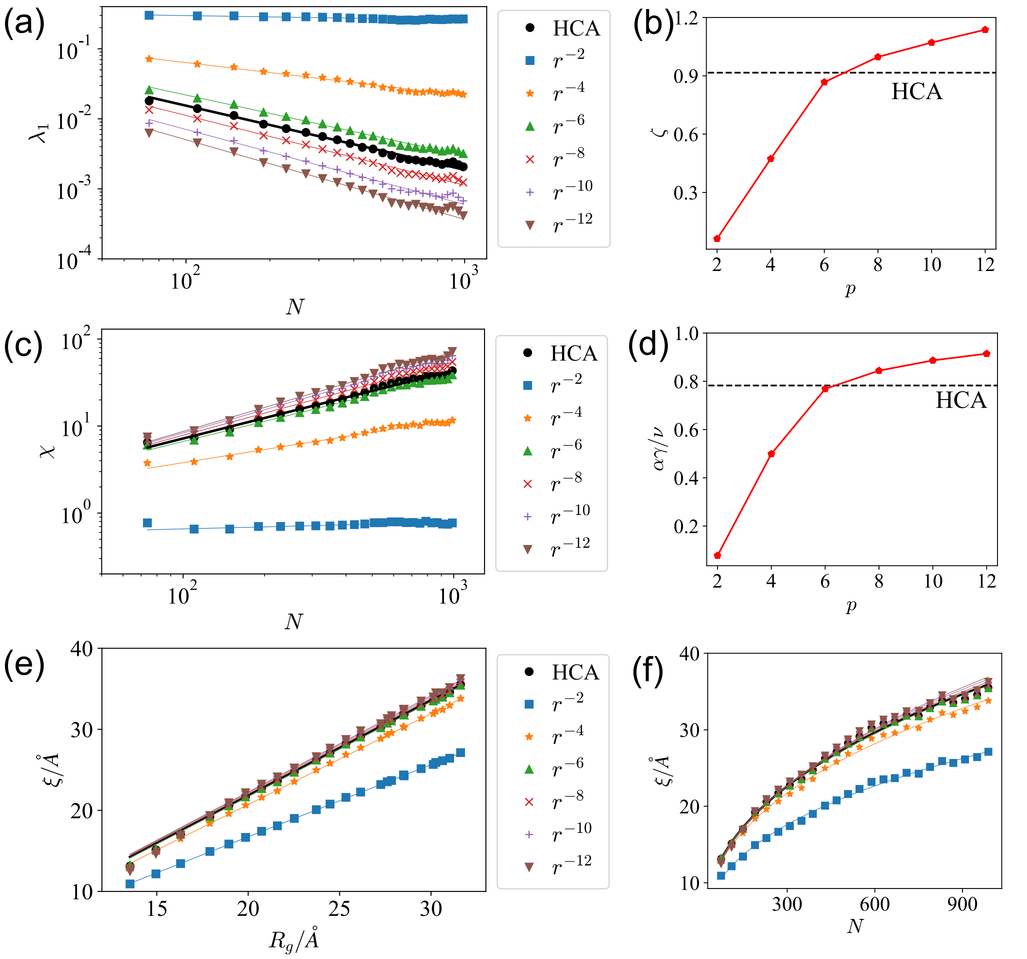


**Figure C. The protein dynamics predicted by the refined versions of elastic network model (HCA model and pfGNM).** (a) The power law relations between the slowest-mode eigenvalue $\lambda_1$ and the chain length $N$. (b) For different models, the critical exponents $\zeta$. The black dash line represents the HCA model ($\zeta \approx 0.92$), and the red line represents the pfGNM with different decaying constant $p$. (c) The power law relations between the susceptibility *chi* and the chain length $N$. (d) For different models, the critical exponents $\alpha\gamma/\nu$. The black dash line represents the HCA model ($\alpha\gamma/\nu \approx 0.79$), and the red line represents the pfGNM with different decaying constant $p$. (e) For different models, the correlation length $\xi$ vs. radius of gyration $R_g$. (f) For different models, the correlation length $\xi$ vs. chain length $N$.

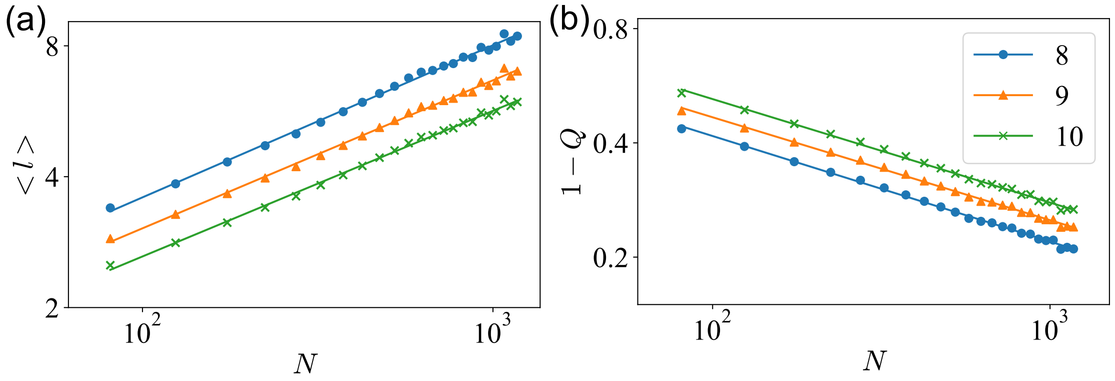


**Figure D. Different cutoff distances $r_c$ shows similar scaling behaviors in the topological descriptors of proteins.** (a) With different cut-off distances ($r_C = 8, 9, 10$Å), the average path length $\langle l \rangle$ vs. chain length $N$ shows similar scaling relation: $\langle l \rangle \sim N^{0.33}$. (b) With different cut-off distances, the relation between modularity $Q$ and chain length $N$ shows similar relation: $1 - Q \sim N^{0.27}$.

$C_\alpha$ atoms of the amino acid residues in proteins are believed to correlate with the vibrational motion of the backbone [10, 11, 12]. In a protein molecule, a high B-factor indicates that the atom exhibits flexibility higher than average, whereas low B-factors are believed to occur at more rigid positions. According to the B-factor profile, information on a lot of biological processes such as ligand binding, catalytic reactions, and protein-protein docking can be gathered [13, 14].

In this section, we neglect the effects of crystal disorder and take B-factor as an indicator of the backbone flexibility. An example is shown in Fig.E(a), in a protein molecule, the residues with low B-factors (colored in red) are usually located at ordered regions of the proteins, or they are deeply buried inside the molecule. The atoms with high B-factors (colored in blue) generally belong to disordered segments such as random coils or flexible linkers exposed at the surface of the protein. Fig.E(b) shows the B-factor profile for every residue in the protein molecule.

Based on the information of B-factors, we evaluate the correlations in a protein molecule. First, we estimate the magnitude of fluctuation of a residue $\langle |\Delta \vec{r}_i| \rangle$ as the square root of the B-factor. Then, one can normalize the magnitude of fluctuation into the Z-score, which is defined by $Z_i = (\langle |\Delta \vec{r}_i| \rangle - \mu)/\sigma$, in which $\mu$ denote for the mean value of the magnitude of fluctuation, and $\sigma$ denote for the standard deviation. In Fig.E(c), the Z-score profile of the normalized magnitude of fluctuations of residues is plotted. Thus, for the Z-score, the mean value fixed as 0, and the standard deviation fixed as 1. Similar normalization has been applied in analyzing and predicting the B-factors of the proteins [15, 16]. With the Z-score profile, we introduce the pairwise correlation $C_{ij}^{(Z)} = Z_i \cdot Z_j$ for residue pair $i - j$. For every protein molecule, one can obtain the correlation matrix $C^{(Z)}$, as shown in Fig.E(d). A positive value of $C_{ij}^{(Z)}$ implies that the fluctuations of residue $i$ and $j$ are both above or below the average, showing positive correlation; while a negative value of $C_{ij}^{(Z)}$ implies that only one of the residues has fluctuation above average, and the other below average. With such a correlation matrix, similar to previous works [4, 17, 18] and our analysis in the main text, the distance-dependent correlation function $C^{(Z)}(r)$ is defined by averaging the correlations for residue pairs at mutual distance $r$. As shown in Fig.E(e), for every protein molecule, based on the correlation matrix, one can obtain the distance-dependent correlation function $C^{(Z)}(r)$. Such a function reflects how the correlations in the magnitude of fluctuations of residue pairs decay with their mutual distances. Further, we define the correlation length $\xi$ as the distance where $C^{(Z)}(r)$ first decays to zero.

As shown in Fig.E(f), for proteins at different sizes, the correlation functions $C^{(Z)}(r)$ exhibit similar behaviors. The correlation functions have high values at short distances, then decay to zero, and continue to decay, showing anticorrelation for residue pairs at relatively long distances. For proteins at different sizes, the correlation functions can be scaled by the $R_g$ of the protein. As shown in Fig.E(g), the scaled correlation functions $C^{(Z)}(r/R_g)$ for proteins of different sizes can

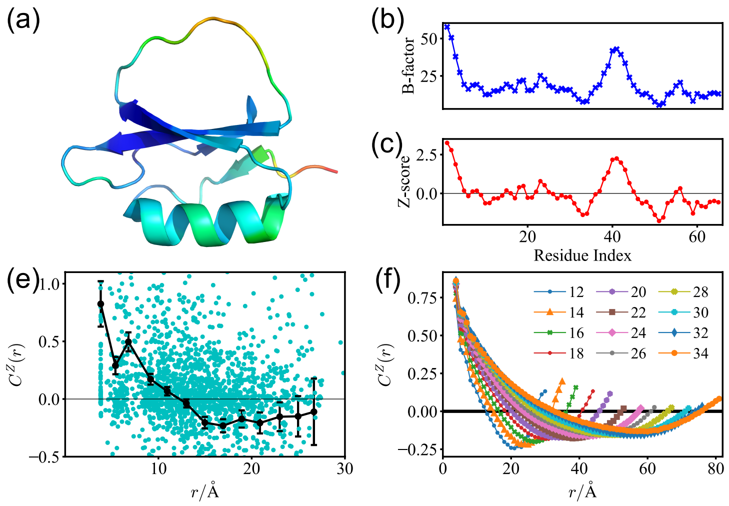

**Figure E. The correlations in the crystallographic B-factors of the proteins.** (a) The illustration of protein structure of the serine proteinase inhibitor CI2 (PDB code: 2CI2, $R_g$ = 12.0 Å) colored by the B-factors of every residue. (b) The B-factors of all the residues in CI2. (c) The Z-scores of all the residues in CI2. (c) The pairwise correlation matrix $C^{(Z)}$ of the protein CI2. (e) The scattering plot (cyan) of the pairwise correlation $C_{ij}^{Z}$ versus inter-residue distance $r_{ij}$ and the distance dependent correlation function $C^{(Z)}(r)$ of the protein CI2 (black), where the dots denote the mean value of the correlation for residue pairs at distance $r$ and the errorbars denote the standard error the mean. The red arrow shows the correlation length $\xi$. (f) The averaged correlation function $C^{(Z)}(r)$ for proteins with different radius of gyration $R_g$. (g) The normalized correlation function $C^{(Z)}(r/R_g)$.

collapse well, indicating that the correlation length $\xi$ of the proteins is proportional the radius of gyration $R_g$ of the proteins. The perfectly collapse of the correlation functions $C^{(Z)}(r/R_g)$ provides additional evidence to the long-range correlations of proteins. It is worth noting that for protein molecules, the high flexibility of residues at the surface boundary region may also contribute to the decaying behavior of such a correlation function.

## References


[1] Moret MA, Zebende GF. Amino acid hydrophobicity and accessible surface area. Phys Rev E. 2007; 75(1): 011920.

[2] Reuveni S, Granek R, Klafter J. Proteins: coexistence of stability and flexibility. Phys Rev Lett. 2008; 100(20): 208101.

[3] Phillips JC. Fractals and self-organized criticality in proteins. Physica A. 2014; 415: 440-448.

[4] Tang QY, Zhang YY, Wang J, Wang W, Chialvo DR. Critical Fluctuations in the Native State of Proteins. Phys Rev Lett. 2017; 118(8): 088102.

[5] Flechsig H. Design of elastic networks with evolutionary optimized long-range communication as mechanical models of allosteric proteins. Biophys J. 2017; 113(3): 558-571.

[6] Rubinstein M, Colby RH. Polymer Physics. 1st ed. Oxford University Press; 2003.

[7] Hinsen K. Structural flexibility in proteins: impact of the crystal environment. Bioinformatics. 2007; 24(4): 521-528.

[8] Fuglebakk E, Reuter N, Hinsen K. Evaluation of protein elastic network models based on an analysis of collective motions. J Chem Theor Comp. 2013; 9(12): 5618-5628.

[9] Yang L, Song G, Jernigan RL. Protein elastic network models and the ranges of cooperativity. Proc Natl Acad Sci USA. 2009; 106(30): 12347-12352.

[10] Karplus PA, Schulz GE. Prediction of chain flexibility in proteins. Naturwissenschaften. 1985; 72(4): 212-213.

[11] Parthasarathy S, Murthy MRN. Analysis of temperature factor distribution in high-resolution protein structures. Protein Sci. 1997; 6(12): 2561-2567.

[12] Moelbert S, Emberly E, Tang C. Correlation between sequence hydrophobicity and surface-exposure pattern of database proteins. Protein Sci. 2004; 13(3): 752-762.

[13] Sun Z, Liu Q, Qu G, Feng Y, Reetz MT. Utility of B-factors in protein science: interpreting rigidity, flexibility, and internal motion and engineering thermostability. Chem Rev. 2019; 119(3): 1626-1665.

[14] Bahar I, Lezon TR, Yang LW, Eyal E. Global dynamics of proteins: bridging between structure and function. Annu Rev Biophys. 2010; 39: 23-42.

[15] Smith DK, Radivojac P, Obradovic Z, Dunker AK, Zhu G. Improved amino acid flexibility parameters. Protein Sci. 2003; 12(5): 1060-1072.

[16] Yuan Z, Bailey TL, Teasdale RD. Prediction of protein B-factor profiles. Proteins. 2005; 58(4): 905-912.

[17] Cavagna A, Cimarelli A, Giardina I, Parisi G, Santagati R, Stefanini F, et al. Scale-free correlations in starling flocks. Proc Natl Acad Sci USA. 2010; 107(26): 11865-11870.

[18] Attanasi A, Cavagna A, Del Castello L, Giardina I, Melillo S, Parisi L, et al. Finite-size scaling as a way to probe near-criticality in natural swarms. Phys Rev Lett. 2014; 113(23): 238102.
