# Supplementary material for: Long-range correlation in protein dynamics: Confirmation by structural data and normal mode analysis
Source: PLoS Comput Biol. 2020 Feb 13;16(2):e1007670. doi: 10.1371/journal.pcbi.1007670 (PMC7043781; doi:10.1371/journal.pcbi.1007670)

Supplementary Code

Qian-Yuan Tang, Kunihiko Kaneko

1 Processing PDB Files

In this section, by taking the crystallized strcuture of the protein serine proteinase inhibitor (PDB code: 2CI2) as an example, we will:

- Read the (x, y, z) coordinates of the C_α atoms.
- Measure the distances between residues r_{ij} and generate the distance matrix.
- Measure the radius of gyration (R_g) and the lengths of the principle axes L_1, L_2 and L_3.

All the calculations are based on the python package MDtraj [1], which is an open package for the analysis of protein structure and molecular dynamics trajectories.

[1] McGibbon, R. T., et al. (2015). MDTraj: a modern open library for the analysis of molecular dynamics trajectories. Biophys J, 109(8), 1528-1532.

[1]:
```
import mdtraj as md
import numpy as np
import matplotlib.pyplot as plt
```

1.1 Load PDB file and Select Carbon-alpha atoms

[2]:
```
# Load PDB file, select carbon-alpha atoms and get the coordinates of these atoms
pdb = md.load_pdb('2ci2.pdb')
residue = pdb.topology.select('name CA')
coord = pdb.xyz[0]
calpha = np.array([coord[i] for i in residue])
calpha *= 10. # From nanometer to Angstrom

# Visualize the 3D structure of the carbon-alpha atoms
from mpl_toolkits import mplot3d
from matplotlib import rcParams
rcParams['font.size'] = 12
fig = plt.figure()
ax = plt.axes(projection="3d")
ax.plot3D(calpha[:,0],calpha[:,1],calpha[:,2],c='darkcyan')
ax.scatter3D(calpha[:,0],calpha[:,1],calpha[:,2],c='navy')
plt.savefig('FigSC1-Calpha.png', dpi=400)
```

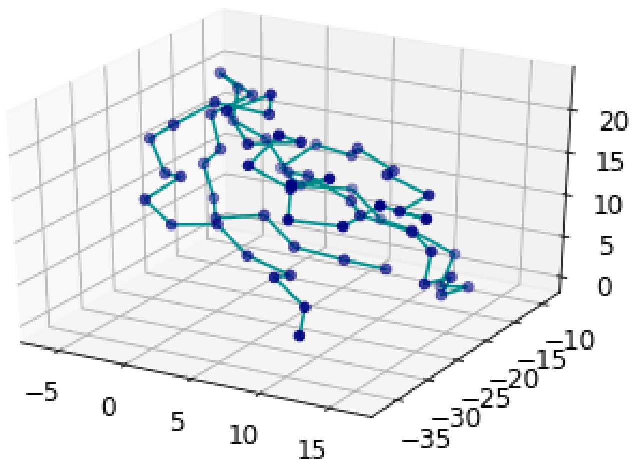

1.2 Generate the distance matrix

[3]:
```
rcParams['font.family'] = 'sans-serif'
rcParams['font.size'] = 18
rcParams['font.sans-serif'] = ['Arial']
plt.subplots_adjust(left=0.18, right=0.9, top=0.9, bottom=0.18)

N = len(calpha)
distance = np.zeros((N,N))
for i in range(N-1):
    for j in range(i+1, N):
        vec = calpha[i] - calpha[j]
        distance[i][j] = (np.dot(vec, vec))**.5
distance += distance.T

plt.title('Distance Matrix')
plt.pcolor(distance)
plt.colorbar()
plt.savefig('FigSC2-Distance.png', dpi=400)
```

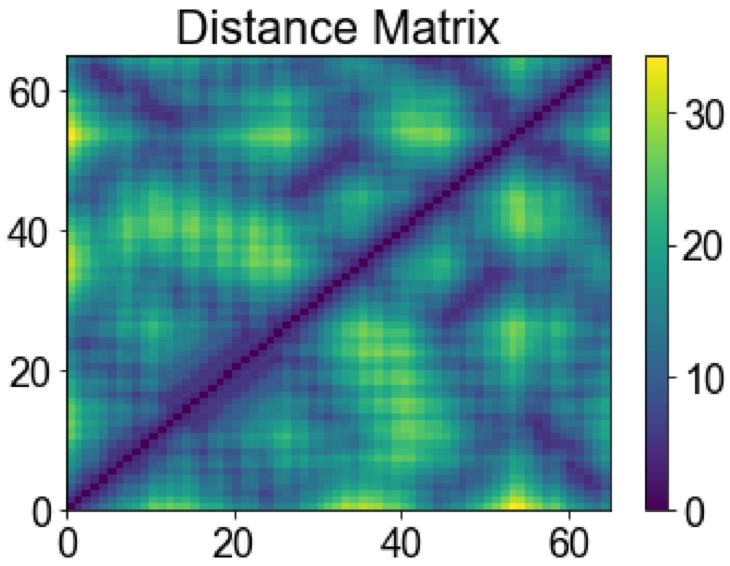

1.3 Describing the Shape of the Protein

Here, the gyration tensor and the principal moments r_1, r_2 and r_3 of the tensor are computed. It is worth noting that only the carbon-alpha atoms are considered in the computation.

The radius of gyration R_g is calculated as: $R_g = \sqrt{r_1^2 + r_2^2 + r_3^2}$.

The shape factor $s = \frac{Na_0^3}{L_1 \cdot L_2 \cdot L_3} = \frac{Na_0^3}{8 \cdot r_1 \cdot r_2 \cdot r_3}$, in which $a_0 = 3.8$Å.

[4]:
```
center = np.mean(calpha, 0)
coord = calpha - center
inertia = np.dot(coord.transpose(), coord)/float(len(coord))
e_values, e_vectors = np.linalg.eig(inertia)
r1, r2, r3 = 0., 0., 0.
eval1, eval2, eval3 = 0., 0., 0.
for i in range(len(e_values)):
    # the largest eigenvalue
    if e_values[i] == max(e_values):
        eval1 = e_values[i]
        axis1 = e_vectors[:,i]
    # the smallest eigenvalue
    elif e_values[i] == min(e_values):
        eval3 = e_values[i]
        axis3 = e_vectors[:,i]
    # the middle eigenvalue
    else:
        eval2 = e_values[i]
        axis2 = e_vectors[:,i]
```

```
# compute the radius of gyration
r1, r2, r3 = eval1 ** 0.5, eval2 ** 0.5, eval3 ** 0.5
rg = ((r1 ** 2.)+(r2 ** 2.)+(r3 ** 2.))**0.5

# compute the shape factor
s = (3.8 ** 3 * N) / (8. * r1 * r2 * r3)

# Output gyration tensor
print('Gyration Tensor')
print(inertia)

# Output the radius of gyration
print('\n R_g = ' + str(round(rg,4)))

# Output the shape factor
print('\n s = ' + str(round(s,4)))
```

```
Gyration Tensor
[[ 41.670933  -12.257082  -14.271482 ]
 [-12.257082   46.304596   -5.8159437]
 [-14.271482   -5.8159437  32.023014 ]]

 R_g = 10.9544

 s = 2.1229
```

2 Elastic Network Model and Normal Mode Analysis

In this section, we will:

- Build the Kirchhoff matrix of the Gaussian network model.
- By conducting normal mode analysis, the oscillation spectrum of the protein can be obtained.
- Predict the cross-correlation matrix, calculate the distance-dependent correlation function and measure the susceptibility and correlation length of the protein.

2.1 Build the Kirchhoff matrix

Here, in the calculation, we set cut-off distance $r_C = 8\text{Å}$.

[5]:
```
r_cut = 8.
Kirchhoff = np.zeros((N, N))

for i in range(N-1):
    for j in range(i+1, N):
        if distance[i][j]<r_cut:
            Kirchhoff[i][j] = -1
            Kirchhoff[j][i] = -1
```

```
            Kirchhoff[i][i] += 1
            Kirchhoff[j][j] += 1
```

2.2 Obtain the oscillation spectrum

Visualize the first 10 eigenvalues of the spectrum:

[6]:
```
plt.subplots_adjust(left=0.18, right=0.9, top=0.9, bottom=0.18)

eigs = np.linalg.eigvalsh(Kirchhoff)[1:] # The zeroth mode is removed

# Normalize the eigenvalues
sumeigs = sum(eigs)
eigs = eigs / sumeigs * float(N)

for i in range(1, 11):
    ratio = eigs[i]/eigs[0]
    plt.plot([0, 1], [ratio, ratio], 'k-', lw=2)
plt.xticks([])
plt.ylabel('$\lambda_i/\lambda_1$')
plt.savefig('FigSC3-eigenvalues.png', dpi=400)
```

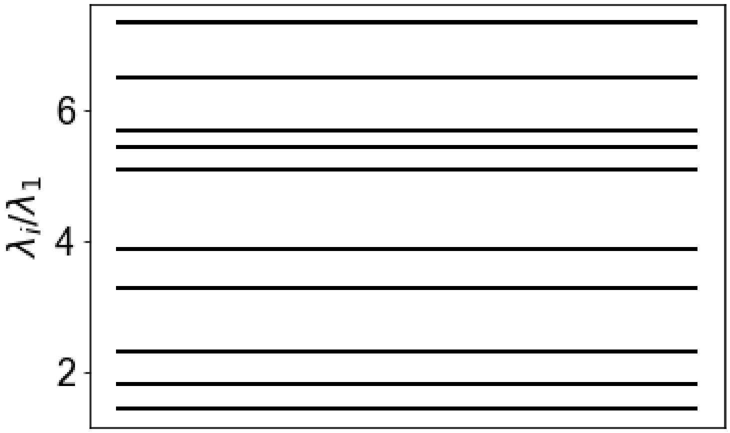

2.3 Compute the covariance and the cross-correlation matrix

[7]:
```
plt.subplots_adjust(left=0.18, right=0.9, top=0.9, bottom=0.18)

Cov = np.linalg.pinv(Kirchhoff)
Corr = np.zeros((N, N))
for i in range(N-1):
    for j in range(i+1, N):
        Corr[i][j] = Cov[i][j]/((Cov[i][i] * Cov[j][j])**.5)
```

```
Corr += Corr.T
plt.title('Cross-correlation')
plt.pcolor(Corr, cmap='coolwarm')
plt.colorbar()
plt.savefig('FigSC4-crosscorrelation.png', dpi=400)
```

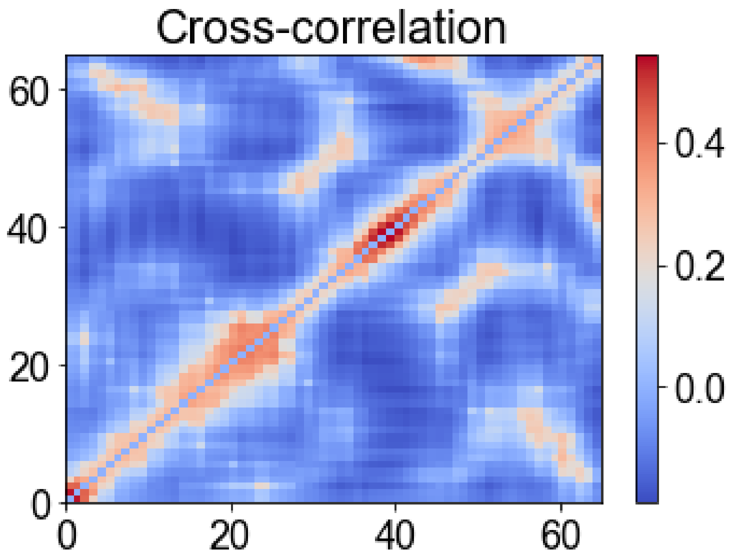

2.4 Compute the averaged distance-dependent correlation function

[8]:
```
d_list = []
c_list = []

d_dict = {}
c_dict = {}

for i in range(N-1):
    for j in range(i+1, N):
        c_list.append(Corr[i][j])
        d_list.append(distance[i][j])
        intd = int(d_list[-1] + .5)
        if not intd in d_dict:
            d_dict[intd] = []
            c_dict[intd] = []
        d_dict[intd].append(d_list[-1])
        c_dict[intd].append(c_list[-1])

d_ave = []
c_ave = []
```

```
for intd in range(200):
    if intd in d_dict:
        if len(d_dict[intd])>10:
            d_ave.append(np.mean(np.array(d_dict[intd])))
            c_ave.append(np.mean(np.array(c_dict[intd])))
```

- Scattering plot of the distance-dependent correlation (Cyan).
- Plot the averaged distance-dependent correlation function $\phi(r)$ (Blue).

[9]:
```
plt.subplots_adjust(left=0.18, right=0.9, top=0.9, bottom=0.18)
plt.plot([0, 35], [0, 0], 'k-', lw=1.5)
plt.plot(d_list, c_list, 'c.', alpha=.3)
plt.plot(d_ave, c_ave, 'bo-', lw=3)
plt.xlim(2, 28)
plt.ylim(-.2, .4)
plt.title('Distance-dependent function')
plt.xlabel('Distance $r$')
plt.ylabel('$\phi(r)$')
plt.savefig('FigSC5-distdependent.png', dpi=400)
```

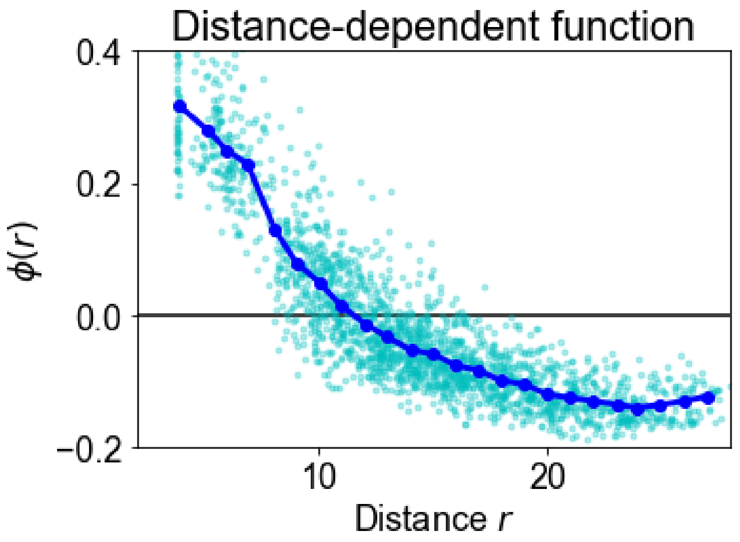

2.5 Compute the correlation length and the susceptibility

For protein systems, we define the correlation length ξ as the distance where correlation function $\phi(r)$ first decays to zero.

The susceptibility χ is defined as the total correlation in a unit volume within the correlation length: $\chi = \frac{s}{N} \sum_{i<j} \phi_{ij} \cdot \theta(\xi - r_{ij})$, where ϕ_{ij} denotes the cross correlation for residue i and j, s denotes the shape factor of protein, and $\theta(x)$ denotes the Heaviside function.

[10]:
```
slope = 0.
for i in range(len(c_ave)-1):
    if c_ave[i]*c_ave[i+1]<0.:
        slope = (c_ave[i]-c_ave[i+1])/(d_ave[i]-d_ave[i+1])
        break

corr_length = round(d_ave[i] - c_ave[i]/slope, 5)
print('Correlation Length = ', corr_length)

sus = 0.
for i in range(N-1):
    for j in range(i+1, N):
        if distance[i][j]<corr_length:
            sus += Corr[i][j]
sus = round(s*sus/float(N), 5)
print('Susceptibility = ', sus)
```

```
Correlation Length =  11.49552
Susceptibility =  2.96641
```

3 Topological Analysis

In this section, topological analysis of the elastic network is conducted. Our analysis is based on the following two packages:

1. **NetworkX** [2]: A Python package for study of the topology and dynamics of complex networks.
2. **Community API** [3]: Compute the partition of the graph nodes which maximises the modularity.

[2]. Hagberg A.A., Schult D.A., Swart P.J. (2008). Exploring network structure, dynamics, and function using NetworkX, in Proceedings of the 7th Python in Science Conference (SciPy2008).

[3]. Blondel V.D., Guillaume J.L., Lambiotte R., Lefebvre E. (2008). Fast unfolding of communities in large networks. J Stat Mech. 2008(10), P10008.

First, we create an empty network with no nodes or edges.

[11]:
```
import networkx as nx
G = nx.Graph()
```

Then, we build the graph based on the distance matrix. We set cut-off distance $r_C = 8$Å.

[12]:
```
for i in range(N):
    G.add_node(i)
for i in range(N-1):
    for j in range(i+1, N):
        if distance[i][j]<r_cut:
            G.add_edge(i, j)
```

```
# Visualize the network
nx.draw_spring(G)
plt.savefig('FigSC6-topology.png', dpi=400)
```

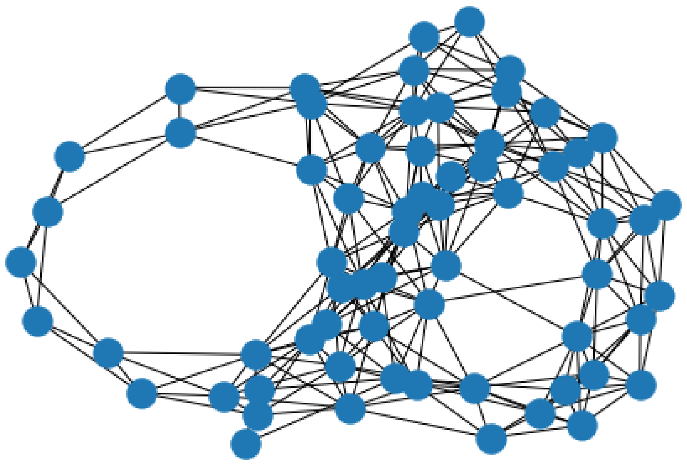

Compute the average shortest path length of the graph:

[13]:
```
ave_path_length = nx.average_shortest_path_length(G)
print("Average Path Length = ", round(ave_path_length,5))
```

```
Average Path Length =  2.90577
```

Finally, we compute the partition of the graph (illustrated below) which maximizes the modularity Q.

[14]:
```
import community

partition = community.best_partition(G)
Q = community.modularity(partition, G)
print('Modularity = ', round(Q,5))

colorlist = ['tab:blue', 'tab:orange', 'tab:green', 'tab:red', 'tab:purple',␣
↪'tab:brown', 'tab:pink', 'tab:gray']
color_map = []
for node in G:
    color_map.append(colorlist[partition[node]])

nx.draw_spring(G, node_color = color_map)
plt.savefig('FigSC7-modularity.png', dpi=400)
```

```
Modularity =  0.49452
```

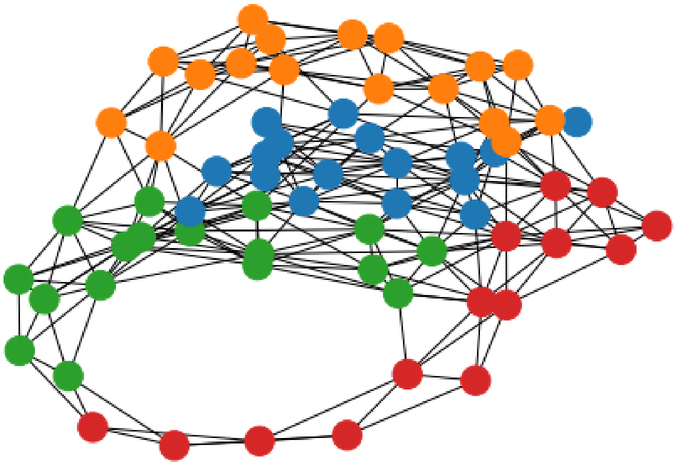

Supplement: S2 Appendix — The code (written in Python language) for PDB file processing, correlation analysis, normal mode analysis, and topological analysis are listed in Supplementary Code. (PDF) [file pcbi.1007670.s002.pdf]
