# Supplementary material for: Long-range correlation in protein dynamics: Confirmation by structural data and normal mode analysis
Source: PLoS Comput Biol. 2020 Feb 13;16(2):e1007670. doi: 10.1371/journal.pcbi.1007670 (PMC7043781; doi:10.1371/journal.pcbi.1007670)
Supplement: Attachment — Submitted filename: Response.pdf [file pcbi.1007670.s007.pdf]

Reviewer #1

The paper presents a large-scale analysis of protein dynamics primarily using GNMs. The work appears to have been carried out carefully and the paper is well-written and understandable.

(1) The results reveal a scaling behaviour that appears to be unique to proteins and these results would be of interest to a certain group of scientists although perhaps not many biologists. I recommend that more is written about how these results can impact on our understanding of protein function and how they might help a biologist investigating a particular system.

We thank the reviewer for the careful reading of the manuscript and helpful comments and suggestions. Now, in the *Results* part, an additional section "*Stability-Functionality Constraint: The size dependence of proteins' shape*" is added. By taking three proteins as examples, our results suggest that the balance between stability and flexibility acts as an evolutionary constraint for proteins at different sizes. These results may help a biologist to design proteins.

(2) I appreciate the GNM part of the paper and have no issues with it. However, I do have issues with the part of the paper that using B-factors and indeed I think it is flawed. The authors suggest that those residues with a positive product of Z values are correlated and those with a negative are anticorrelated. They then look at the distance behaviour of the correlation and see that it scales according to the radius of gyration. This shows a "scale-free" behaviour. My interpretation would be different. The correlation distance is not a correlation distance but a distance that relates to a surface boundary region where there is a transition between residues with low B-factors (internal) and to a region where residues have high B-factors (on or near the surface). It is not surprising therefore that this scales with the size of the protein. Can the authors rule out this explanation? If so they should and if not they might consider removing this section and rely instead on the GNM results alone.

The idea of measuring the correlations in magnitudes comes from neurosciences. In brain research, one may be interested in the brain areas that show high correlations in their activity. However, for protein molecules, the high flexibility of the surface boundary region becomes a very important effect. Therefore, we cannot rule out the explanation pointed out by the reviewer. Now,

we have put our discussions based on B-factor analysis into the Supplementary Materials, by remarking on the possible limitation. Our discussions in the main text now only rely on the GNM results.

(3) I found the paragraph before the Conclusion section long-winded. The authors should consider shortening it.

We thank the reviewer for pointing out this. Now we have revised the manuscript accordingly.

Reviewer #2

This article presents a network-centric analysis of collective motions in proteins, based on two approaches: (1) the analysis of crystallographic B-factors and (2) the analysis of Elastic Network Models constructed from crystallographic protein structures. They compare protein-derived networks with random networks and interaction networks of lattice structures. Their findings are interesting, shedding new light on phenomena that have been known empirically for a long time. They also seem novel to me, but I may be unaware of similar prior work because I have not followed network-centric approaches specifically over the last few years. For the same reason, I have not been able to verify many specific assertions on network properties made in the article.

(1) My main criticism of this work is that it is not reproducible. No statement is made about the algorithms applied and software used. No references to published software, no project-specific software available as supplementary material. Unfortunately, the PLOS policy on software sharing is weak and unrealistic (https://journals.plos.org/ploscompbiol/s/materials-and-software-sharing), so the lack of software looks compatible wit this policy, but as a reviewer I have to say that it makes it impossible for me to verify the results of this submission.

We thank the reviewer for valuable and constructive comments and suggestions. In this submission, the code and the data are attached so the reviewers and readers can verify and reproduce our analysis.

General comments:

(2) The authors refer to critical behavior in many places. This term has different (though related) meanings in different disciplines. The authors' use of the term best fits the concept of self-organized criticality in my opinion (which is also suggested by the titles of references 17 to 20). The authors should then say this in the article (or add some other clarifiation if they don't agree with mine). The authors should also explain in much more detail how their work relates to the earlier studies on self-organized criticality in proteins they refer to.

Our analysis focuses on these critical behaviors such as the long-range correlations and scaling relations (power-laws) in protein systems. And self-organized criticality (SOC) is a mechanism that attempts to explain why so many complex systems exhibit power-laws and other critical behaviors.

In the references 17-20, the authors focus on the hydrophobicity scale of amino acids. They found a difference between a hydrophobicity scale with SOC effect taken into account (MZ scale) and the conventional hydrophobicity scale. It was concluded that such a difference reflects the “long-range conformational forces” that are central to protein functionality. Such “long-range conformational forces” are in line with the long-range correlations discussed in our manuscript. In the revised version, additional explanations are added to show such a connection.

(3) The choice of random networks and lattice structures as references for comparison looks a bit arbitrary. Random networks do not correspond to any interaction network in physical systems. Lattices do, but they are very far from proteins in terms of physical properties. The most interesting systems to compare to, in my opinion, are non-biological soft matter systems, such as polymers.

In this version, we update our discussions by comparing proteins with lattices and polymer chains. All our figures and discussions are updated accordingly.

(4) The authors make specific predictions, e.g. on the scaling of the slowest modes with protein size, that should be amenable to experimental validation. Have they searched for experimental studies in the literature?

In fact, we tried to search for experimental studies that can directly validate our study, but most experiments only focus on the dynamics of a specific kind or family of proteins. There are no experiments that span over proteins at all different sizes and measure their vibrations in a very similar environment.

Although there are no direct experimental data, we can provide indirect evidence to support our scaling results. Since the modes are encoded in the structure of a protein, we can check the relations between the shape (structure) and the size instead in order to validate the relation between modes and size. Now in the *Results* part, an additional section “*Stability-Functionality Constraint: The size dependence of proteins' shape*” is added.

Specific comments by page number:

(5) Page 2: "Although there are only short-range physical interactions among the residues,"

Residues being charged, there are long-range interactions as well. It is true however that models that leave out the long-range interactions (e.g. ENMs) also exhibit long-range correlations, suggesting that the long-range interactions are perhaps not essential.

We agree with the reviewer. Now this sentence was deleted. Further discussions about the interaction range and correlation length are added to the discussions on page 4 and 5 in the *Results* part.

(6) "B-factors"

The authors repeat a popular false assumption in the study of protein dynamics: the idea that B-factors measure thermal fluctuations. In crystallographic structure refinement, a distribution of conformations is fitted to the observed Bragg peaks. This distribution is most commonly described by a Gaussian model, consisting of an average structure and a variance. The variance matrix is usually approximated by a diagonal matrix, whose non-zero elements are the B-factors. B-factors thus measure all deviations from an ideal crystal at temperature zero: finit-size effects, crystal disorder, and thermal fluctuations. Crystallography studies of the same protein at different temperatures (e.g. PDB codes 1IEE and 2LYM, both for tetragonal lysozyme) show that the influence of temperature on B-factors is very small, suggesting that the dominant contribution is crystal disorder.
This doesn't invalidate the authors' analysis, as crystal disorder effects also spread through the protein via inter-residue contacts. It is only the presentation in terms of conformational fluctuations that needs to be revised.

Page 4: "X-ray diffraction can only provide one static structure"
See above. An X-ray structure is not static, it is the average structure in a Gaussian ensemble.

Page 5: "From the B-factor profile, one can estimate protein flexibility, "
This looks dubious. As said above, B-factors don't really measure fluctuations. And even if they did, it is not obvious how fluctuations at the atomic level, without inter-atomic conformational correlations, can be translated into some measurer of flexibility.

Page 5: "A positive value of C^{(Z)}_ij" implies that the fluctuations of residues i and j are both above or below the average..."
Why is this relevant? Does the average have any scientific interpretation that makes above/below average meaningful?

We thank very much the reviewer for pointing out our misunderstandings on X-ray diffraction and B-factors. Now in the revised version, we corrected all our statements.

As mentioned in the response to Reviewer 1, the idea of measuring the correlations in magnitudes originated from neurosciences. In brain research, one may be interested in the brain areas that show high correlations in their activity. However, for protein molecules, similar methods may not be suitable because there is always high flexibility for residues at the surface boundary region. Now we put our discussions based on B-factor analysis into the Supplementary Materials, by remarking on the possible limitation.

(7) Page 7: "it can be concluded that the collective motions of residues and the critical fluctuations of native proteins are encoded in the native structures"
The encoding of collective motions in the structure is the fundamental hypothesis of Elastic Network Models, so it cannot be concluded from an analysis of their results. The added value contributed by the authors is a more specific characterization of these dynamics in comparison to other types of networks.

Indeed, the encoding of collective motions in the structure is the fundamental hypothesis of Elastic Network Models. We have updated related discussions.

In fact, before our research, no one had answered the question that whether all the long-range correlations can be successfully modeled by ENMs. Our paper wants to show that with only local interactions, ENM can already reproduce the long-range correlations and critical dynamics of the proteins.

(8) Page 8: "Slow modes and critical fluctuations of native proteins"
The authors use the simplest form of ENM, in which the force constant for each residue pair can only take the values 0 or 1. It has been shown in the past (see e.g. http://doi.org/10.1021/ct400399x) that these models describe the "real" protein dynamics (obtained from experiment or from more detailed models) rather badly and that ENMs with distance-dependent force constants yield better matches. This raises the question if the mode frequency scaling behavior observed by the authors also holds if more accurate protein models are used.

The reviewer had pointed out an important question. To solve this problem, we conducted a similar analysis with some different kinds of ENMs with distance-

dependent force constants. Some of the discussions are added to the main text, and the detailed results are listed in Supp. Info.

It is observed that the simplest form of ENM can have similar scaling relations with the harmonic C-alpha potential model (HCA). Such a result shows that the eigenvalue distributions in protein molecules are quite robust.

However, with an inverse square law, pfGNM fails to predict all the scaling relations in the proteins. Previous researches already found that pfGNM can only be applied for proteins in crystalline conditions, and it will have a poor agreement to the collective motions given by molecular dynamics.

(9) Page 9: "Noting that the dynamics are encoded in the structures, that is to say, the structures of proteins, which are optimized through the process of molecular evolution, are significantly different from the regular 3D lattice structures."
If the goal is to show the difference between biologically evolved systems and simpler non-adaptive physico-chemical systems, the comparison should not be 3D lattices but non-biological soft matter, e.g. polymers.

As we have mentioned in Question 3, in this revised version, we update our discussions by comparing proteins with lattices and polymer chains. All our figures and discussions are updated accordingly.
